# Non-Contact Sensor for Long-Term Continuous Vital Signs Monitoring: A Review on Intelligent Phased-Array Doppler Sensor Design

**DOI:** 10.3390/s17112632

**Published:** 2017-11-15

**Authors:** Travis Hall, Donald Y. C. Lie, Tam Q. Nguyen, Jill C. Mayeda, Paul E. Lie, Jerry Lopez, Ron E. Banister

**Affiliations:** 1 Department of Electrical and Computer Engineering, Texas Tech University, Lubbock, TX 79409-3102, USA; travis.hall8908@gmail.com (T.H.); tam.nguyen@ttuhsc.edu (T.Q.N.); jill.mayeda@ttu.edu (J.C.M.); jerry@noisefigure.com (J.L.); 2 Texas Tech University Health Sciences Center, Texas Tech University, Lubbock, TX 79430, USA; plie@usc.edu (P.E.L.); ron.banister@ttuhsc.edu (R.E.B.)

**Keywords:** active antenna, beam-steering, Doppler Radar, e-health, homecare, noncontact vital signs monitoring, phased array, intelligent sensor, telemedicine, vital signs monitoring, wireless assisted living, wireless acute care

## Abstract

It has been the dream of many scientists and engineers to realize a non-contact remote sensing system that can perform continuous, accurate and long-term monitoring of human vital signs as we have seen in many Sci-Fi movies. Having an intelligible sensor system that can measure and record key vital signs (such as heart rates and respiration rates) remotely and continuously without touching the patients, for example, can be an invaluable tool for physicians who need to make rapid life-and-death decisions. Such a sensor system can also effectively help physicians and patients making better informed decisions when patients’ long-term vital signs data is available. Therefore, there has been a lot of research activities on developing a non-contact sensor system that can monitor a patient’s vital signs and quickly transmit the information to healthcare professionals. Doppler-based radio-frequency (RF) non-contact vital signs (NCVS) monitoring system are particularly attractive for long term vital signs monitoring because there are no wires, electrodes, wearable devices, nor any contact-based sensors involved so the subjects may not be even aware of the ubiquitous monitoring. In this paper, we will provide a brief review on some latest development on NCVS sensors and compare them against a few novel and intelligent phased-array Doppler-based RF NCVS biosensors we have built in our labs. Some of our NCVS sensor tests were performed within a clutter-free anechoic chamber to mitigate the environmental clutters, while most tests were conducted within the typical Herman-Miller type office cubicle setting to mimic a more practical monitoring environment. Additionally, we will show the measurement data to demonstrate the feasibility of long-term NCVS monitoring. The measured data strongly suggests that our latest phased array NCVS system should be able to perform long-term vital signs monitoring intelligently and robustly, especially for situations where the subject is sleeping without hectic movements nearby.

## 1. Introduction

In this paper, we will first cover why non-contact vital signs (NCVS) sensor monitoring systems should be of great importance and interests to study and research and present some examples of existing NCVS sensors. After that we will discuss the design methodology behind our own Doppler-based NCVS sensor systems and focus on our NCVS sensor systems development as they became more powerful over time. Lastly, we will present the test results on our design prototype of an intelligent phased-array Doppler sensor that can perform automatic beam-steering to track the subject to enable long-term continuous heart and respiration rates monitoring.

### 1.1. Research Motivation

Vital signs data, such as of blood pressure, temperature, respiration rate and heart rate, is one of the most important factors in patient’s care and diagnosis as it allows physicians and healthcare professionals to make decisions regarding a patient’s treatment options and well-being. In most cases, blood pressure and temperature only need to be taken once every few hours, while respiration and heart rates are often continuously monitored for patients in the ER (emergency room) or in an ICU (intensive care unit). Because of this difference of monitoring needs, we have been developing our custom non-contact vital signs (NCVS) sensor by focusing on its capturing and recording of the respiration rates and heart rates continuously and accurately.

Most methods of measuring respiration and heart rates involve physical contacts with the subject. Contact-based vital signs sensors include pulse oximeters, ECGs (electrocardiograms) measurement systems with electrodes, piezoelectric sensors and even implantable cardiac sensors that take IEGMs (intra-cardiac electrograms) as the pacers, as well as the ICDs (implantable cardiovascular defibrillators). Many companies, such as Fitbit (https://www.fitbit.com/), isansys (http://www.isansys.com/) and Sotera Wireless (http://www.soterawireless.com/), to name a few, have developed sensors that physically contact the patients but wirelessly transmit the vital signs information to a computer, tablet, or phone to eliminate the wires between a patient and the monitoring system. Many of these physical sensors with wireless capabilities, however, suffer from the limitation of finite battery life. For continuous vital signs measurements, a physical contact-based wireless system requires a battery with a large capacity; and in some cases, the battery reaching the end of its charge can also result in the inaccuracy of vital signs measurement.

Unfortunately, it is not always possible to use these contact-based sensors for patients’ vital signs monitoring, especially when dealing with burn victims or infants. Newborns requiring an intensive care unit (ICU) often suffer skin damage as a result of using electrode contacts, adhesive tape and other probes to measure vital signs. In some cases, using these physical measurement devices result in physical scars on the newborn child. For burn victims, it can be difficult to locate an area with enough uncompromised skin to attach probe. In many instances, an esophageal ECG electrode is used and in severe cases, the electrode has to be stapled to the skin [1]. Using non-contact methods would allow for the measurement of vital signs signals without having to risk further injury to a patient.

Continuous monitoring of patient can be an invaluable tool for doctors and health care professionals, especially useful when considering overnight measurements. An estimated one in five adults suffer from mild obstructive sleep apnea (OSA), or the restriction of airflow despite respiratory efforts, with around one in fifteen adults suffering from moderate OSA. Hypertension, cognitive impairment and increased daytime sleepiness are a few of the negative consequences of OSA. Sudden infant death syndrome (SIDS) is the third leading cause of infant mortality and thought to be linked to sleep apnea. Doppler radar monitoring systems would allow for non-invasive, continuous monitoring of patients and allow for the study and detection of OSA and SIDS, as well as other medical conditions where continuous NCVS is required or beneficial.

### 1.2. Existing Noncontact Vital Signs Sensors

There are several different types of NCVS sensors and some are already available on the market and while some are more academic. I will list the three main types below:

#### 1.2.1. Under-the-Bed Noncontact Sensors for Vital Signs Monitoring

Electronics company muRata (http://www.murata.com/) have created an under-the-bed biomedical system using an accelerometer to capture bed movement caused by respiratory and heart beat movement [2]. The muRata sensor is based on the Ballistocardiograph (BCG) principle. An ultra-sensitive accelerometer is used to capture the vibrations upon the bed caused by a subject’s heart rate, respiration and body movement. A microcontroller is used to process the information, resulting in heart rate, respiration, heart rate variability and stroke volume measurements as well as bed status indication. The company has two product formats; a standalone sensor module and a sensor module with a built-in Wi-Fi transceiver to transmit the vital signs information.

Another company with under-the-bed NCVS products markets the Emfit QS product that has a contact-free sleep tracker device with Wi-Fi connection, connected to under-mattress sensor [3]. It is considered non-contact because the sensor does not contact one’s body as it has a thin strip of electro-sensitive polymer sensor films material that sits under the bed sheet or mattress. The NCVS product can provide detailed sleep reports and 30-Day Trends on an accompanied intuitive web app, that reports the whole night Heart-Rate-Variability (HRV), RMSSD (Root Mean Square of the Successive Differences) of HRV, detailed heart & breathing rates, sleep score, resting heart rate, etc. in either REM (rapid eye movement) or light sleep stages. The long term HRV monitoring can indicate if one’s cardiovascular fitness is increasing, as baseline HRV increases along with it. The short term HRV gives valuable information about stress and recovery, as both tend to decrease HRV from the baseline. This sensor technology is used for measuring basic physiology (e.g., heart rate, respiration rate, movement activity) passively, from below the patient’s mattress, without any electrodes, leads, cuffs or cannula. The sensor technology can be used for adults and children in various care environments but no clinical data is available from its website. However, since this kind of under-the-bed NCVS technology requires the embedded polymer films or accelerometers to interpolate its pressure data and therefore the heart rates, it seriously limits its practical use for most of the NCVS monitoring in the office or at home while objects sitting on an office chair or a sofa. It may also be challenging for this technology to be able to track the vital signs of two people accurately who sleep on the same mattress at the same time (e.g., husband and wife), as the extra motion from the other person on the mattress can cause considerable errors on HRV extraction, etc. The firmness of the mattress and the thickness and flatness of the bedsheets and blankets may affect the accuracy of this kind of sensors too. It may also be difficult for this kind of pressure-based NCVS sensor to accurately track the vital signs of tiny infants in the neo-natal ICU setting for preventing SIDS, as their weights and lengths and chest movement are considerably smaller.

A group at Kansai University, Osaka, Japan have designed a 24 GHz microwave radar, designed to be placed underneath a mattress, to measure respiration rate and heart rate of patients [4]. The goal of their system was to measure the vital signs signals of elderly patients and detect apneic episodes. While testing seven elder volunteers, with an average age of 93 and eight student volunteers, with an average age of 23, the group was able to successfully detect heart rate, respiration rate and some instances of apnea.

#### 1.2.2. Camera-Based NCVS

Some companies, such as Microsoft, have employed the use cameras to track human movement and detect heart rate without physically contacting a subject. Microsoft’s Xbox One Kinect [5,6] peripheral is a camera system possesses the capability to track a user’s movement through the use of RGB and IR cameras. The Xbox One Kinect sensor was made available for purchase July, 2014 and it was advertised that the heart rate measurement capabilities can be accessed by any developer who wishes develop programs for it [7]. By measuring the change in color of a subject’s face due to heart beat, the Xbox can calculate the heart rate of that subject. This system is not without its limitations though; users must remain fairly still, measurements take a few seconds (at least 5 to 10 s per measurement) to calculate and measurements are not always accurate.

Rice University has received funding from the National Sciences Foundation (NSF) to develop a method for measuring vital signs using photoplethysmography (PPG), an optical measurement of blood volume change in the human body [8]. By measuring change in the amount of hemoglobin (Hb) and oxyhemoglobin (HbO_2_) in any particular area of a subject’s face, the heart rate of that subject can be determined. Using a monochrome camera and their own PPG estimation algorithm, the team at Rice was able to measure heart rate, respiration rate and blood oxygen level of a subject. However, a bit more test data with statistics is needed to show the PPG method is suitable for long-term continuous NCVS monitoring.

A current clinical trial using non-contact measurement of vital signs is being conducted at University of Virginia (UVA) since Nov. 2014 [9]. This study is to test the accuracy of a web cam-based biomedical sensor developed at UVA (not FDA-approved). This trial is designed to measure heart rate, respiratory rate and oxygen saturation (SpO_2_) without requiring any patient contact. One potential application of such a device would be for infant monitoring to allow clinicians and/or parents to monitor the vital signs of infants continuously. The trial proposes to record the vital signs of 100–120 infants (defined as children aged 12 months or less) who are receiving continuous oxygen, heart rate and respiratory rate monitoring with a traditional “gold standard” vital signs monitor (i.e., GE monitoring equipment in this case). Unfortunately, this data of this interesting clinical trial is not available to the public yet for analysis, especially on the long-term continuous monitoring.

A group from the Computer Vision and Image Processing Laboratory at the University of Louisville, Louisville, KY, have used long-wave infrared camera imaging for NCVS monitoring [10]. By using thermography, the study of heat distribution in structures or regions, they were able to measure heart rates and respiration rates of individuals. Using thermal imaging to observe the temperature change at the carotid vessel complex, the group was able to determine the heart rates and respiration rates of four individuals sitting one meter way with 100% accuracy. More data is required to show its practical efficacy.

#### 1.2.3. Doppler-Based NCVS

NCVS sensor products that uses microwave Doppler technologies have also been explored for a long time. The latest summary of advancement in this field has been well-documented in a recent book “Doppler Radar Physiological Sensing” in 2016 [1]. J.C. Lin has reported NCVS monitoring using X-band CW Doppler radar on subjects 30 cm away in 1975 [11]. In 1977, J.C. Lin used the same system, with an additional apnea-detector circuit, to have successfully detected instances of hyperventilation and apnea in rabbits and cats.

Throughout the mid-1980s to late-1990s, radar transceivers began incorporating analog and digital signal processing to separate heart rate movement from respiration movement. Previously, subjects had to hold their breath for a system to be able to detect their heart rate. Advancements in signal processing allowed for the measurement of vital signals through walls and rubble. Recent efforts of NCVS systems have been made to reduce system size, lower power, decrease weight and increase accuracy and robustness. However, the motion artifacts issues during NCVS monitoring were still not fully resolved yet. Recently, Kyoto University Center of Innovation and Panasonic are collaborating to create an NCVS biosensor using millimeter-wave spread-spectrum radar technology [12] and a biomedical company SENSIOTEC have developed an Impulse Radio Ultra-Wideband sensor that is placed underneath a mattress [13]. Both sensors are designed to measure patient heart rate, respiration and monitor movement.

However, even with all of the Doppler-based NCVS system, the authors are not aware of any reported Doppler NCVS system that is intelligent enough to track/follow the patient long-term natural movement on bed to report accurate over-night continuous vital signs data. There is no measured work to demonstrate this possibility until our group published the pioneering work in the past few years, demonstrating the world’s 1st phased-array intelligent Doppler NCVS sensor that performs long-term automatic beam-steering for continuous NVCS on subjects sitting in an office or during sleep [14,15,16]. Therefore, we will detail our work in this specific area of Doppler-based NCVS monitoring next in this paper.

## 2. Research Methodology and Design

The purpose of this short review is to highlight the existing various NCVS systems and the novel phased array beam-steering NCVS system that our lab has pioneered with robust performance developed at the RF-SoC group at Texas Tech University. The idea behind this research was to realize an automatic phased array beam-forming NCVS system that can perform robust long-term vital signs monitoring, for example, during sleep. Therefore, we will show here some of our work in this specific area of NCVS monitoring to demonstrate our novel custom phased-array Doppler NCVS system is robust and intelligent enough to perform long term NCVS monitoring with robustness. In this section, we will cover the mathematical concepts behind the design, as well as a brief history of the development of our NCVS sensor systems.

### 2.1. Our Basic Doppler NCVS Sensor Design Principles

**Figure 1:**
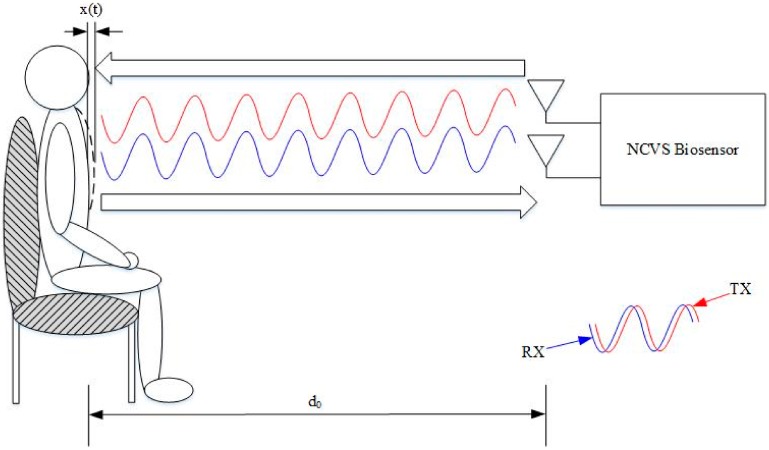
An illustration describing the basic transmitted and reflected electromagnetic waves of the Doppler-based NCVS sensor.

Having a log of continuous vital signs measurement data can be one of the most useful assets a physician has on a patient when making a diagnosis, or it can be very important for a coach/individual before making a work-out training decision. Our NCVS biosensor is a novel form of non-contact vital sign sensor that implements a phased array system in order to measure respiration and heart rate. Our system uses a 2.4 GHz continuous wave (CW) RF signal that is reflected off a subject’s chest wall back to the sensor. The displacement caused by the chest movement (cause by respiration and heart beat) returns a phase-modulated signal to the NCVS sensor that can be translated into an analog vital sign signal. This is demonstrated in Figure 1. The operation principle of the Doppler-based NCVS system can be best understood by looking at this equation:

(1) $$\Phi_{r}\left(t\right)=\;\frac{2f}{c}\left(2\pi x\left(t\right)\right)=\frac{4\pi x\left(t\right)}{\lambda }$$

Φ*_r_*(*t*) is the phase modulated signal used for vital sign measurement, *x*(*t*) represents the chest displacement as a result of a subject’s respiration and heartbeat and *λ* is the wavelength of the transmitted signal. The transmitted signal is defined as:

(2) $$C\left(t\right)=\;A_{T}\cos\left(2\pi ft+\phi \left(t\right)\right)$$

A_T_ is the amplitude of the transmitted signal and ϕ(*t*) is a phase shift caused mostly by VCO phase noise. After being reflected off of a subject’s chest wall, the received signal is defined as:

(3) $$R\left(t\right)=A_{R}\cos\left[2\pi f\left(t-t_{d}\right)+\phi \left(t-t_{d}\right)+\theta \right]$$

A_R_ is the amplitude of the received signal, ϕ(*t*) is the VCO phase shift, *θ* is the constant phase shift and $t_{d}$ represents the time delay of the reflected signal and can be expanded to:

(4) $$t_{d}=\frac{2d\left(t-\frac{d\left(t\right)}{c}\right)}{c}=\frac{2\left(d_{0}+x\left(t-\frac{d\left(t\right)}{c}\right)\right)}{c}$$

Since *x*(*t*), the chest wall displacement, is much smaller than *d*_0_, the distance of the subject from the sensor, the $\frac{2x\left(t-\frac{d\left(t\right)}{c}\right)}{c}$ term can be neglected and the received signal can be approximated as:

(5) $$R\left(t\right)=A_{R}\cos\left[2\pi ft-\frac{4{\pi d}_{0}}{\lambda }-\frac{4\pi x\left(t\right)}{\lambda }+\phi \left(t-\frac{2d_{0}}{c}\right)+\theta \right]$$

To avoid “null” points, areas where a subject’s distance from the sensor is an integer multiple of *λ*/4, causing subject movement to generate a very small amount of phase variation, a quadrature system was employed. The received signal is split into two channels, an In-phase signal and a 90°-offset Quadrature signal. When one channel signal is in a “null” position, the other channel signal is in in an “optimal” position. The *I* and *Q* channels are defined as:

(6) $$I_{B}\left(t\right)=\cos\left[\theta +\frac{4\pi h\left(t\right)}{\lambda }+\frac{4\pi r\left(t\right)}{\lambda }+∆\phi \left(t\right)\right]$$

(7) $$Q_{B}\left(t\right)=\sin\left[\theta +\frac{4\pi h\left(t\right)}{\lambda }+\frac{4\pi r\left(t\right)}{\lambda }+∆\phi \left(t\right)\right]$$

*h*(*t*) is the heart motion signal and *r*(*t*) is the respiration motion signal. Again, *θ* is the constant phase shift and ϕ is the phase shift of the VCO. The *I* and *Q* signals can be combined into a single signal using Arctangent demodulation resulting in:

(8) $$\Phi_{r}\left(t\right)=\tan^{-1}\left(\frac{Q_{B}\left(t\right)}{I_{B}\left(t\right)}\right)=\frac{\sin\left[\theta +\frac{4\pi h\left(t\right)}{\lambda }+\frac{4\pi r\left(t\right)}{\lambda }+∆\phi \left(t\right)\right]}{\sin\left[\theta +\frac{4\pi h\left(t\right)}{\lambda }+\frac{4\pi r\left(t\right)}{\lambda }+\Delta \phi \left(t\right)\right]}\;\;=\left[\theta +\frac{4\pi h\left(t\right)}{\lambda }+\frac{4\pi r\left(t\right)}{\lambda }+∆\phi \left(t\right)\right]$$

**Figure 2:**
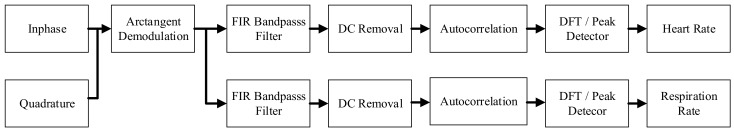
Block diagram of DSPs for extracting vital signs information of our NCVS sensor.

Therefore, from this time-domain signal of the periodic displacement of the heart and the chest wall (i.e., for respiration) displayed in Equation (8), one can extract the heart rate and respiration rate at the same time by applying filtering, DC removal, autocorrelation, DFT (Discrete Fourier Transform) and peak finding of the *h*(*f*) and *r*(*f*) signals in the frequency domain as shown in Figure 2.

Hardware-wise, our phased-array NCVS system utilizes the National Instruments’ (NI) graphical programming language, LabVIEW and an NI USB-6009 Data Acquisition Unit (DAQ) to interface with the NCVS sensor and record vital signs measurements. The NI DAQ digitizes our baseband signal and our LabVIEW program analyzes the data, extracts heart and respiration rates and saves the data into an excel file that can be analyzed later. A custom LabVIEW program was written to capture the vital signs signals. Upon being sampled by the NI DAQ, the in-phase (I) and quadrature (Q) channels are combined through the use of arctangent demodulation [17]. The arctangent signal is duplicated, then split into to two separate signals, one for heart rate and the other for the respiration rate. Each signal is then filtered using a high-order FIR (finite impulse response) bandpass filter. Because the respiration rate is generally quite low in frequency compared to the heart rate, the heart rate signal’s passband is higher than that of the respiration rate. Since the presence of DC offset resulting from receiver imperfections and clutter reflections can be large compared to the ac motion-related signal that we desire, they cannot simply be included in digitization without adversely affecting the resolution [1]. The DC components of these signals are therefore removed. Afterwards, autocorrelation is applied to each signal to help strengthen the periodicity. A discrete Fourier transform (DFT) is performed on each signal to convert them from the time domain to the frequency domain. A peak detector function is used to detect the strongest frequency, which we use as our vital signs measurements.

**Figure 3:**
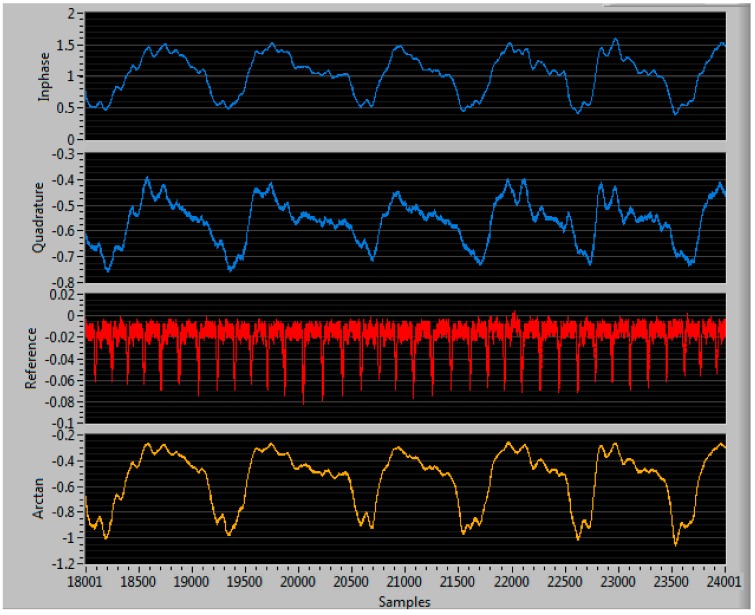
Example of vital signs signals sampled by the NI DAQ.

**Figure 4:**
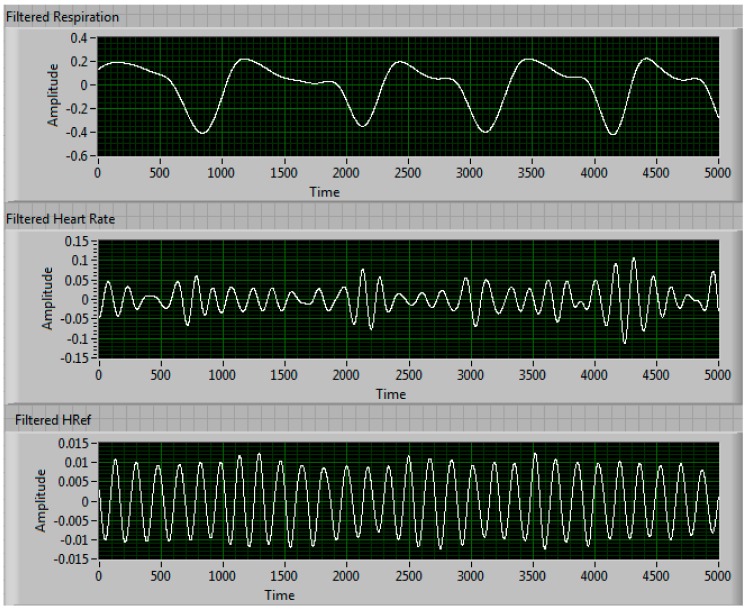
Vital signs signal waveforms after filtering.

**Figure 5:**
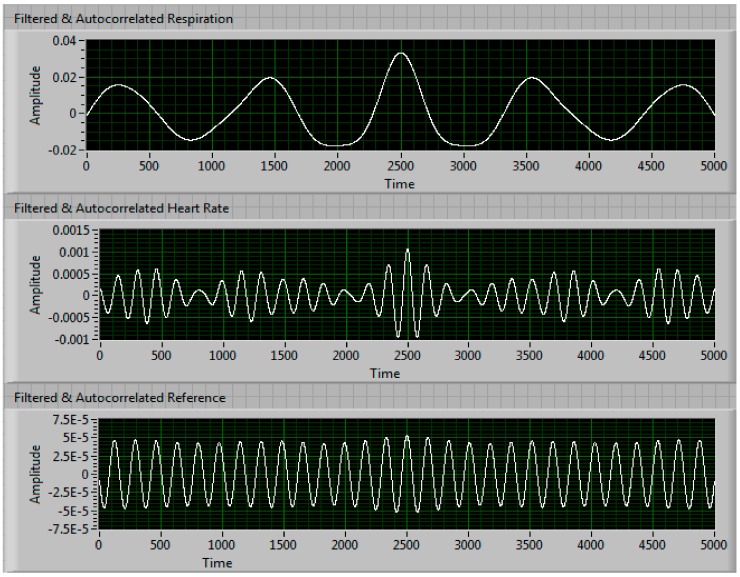
Vital signs signal waveforms after filtration and autocorrelation.

**Figure 6:**
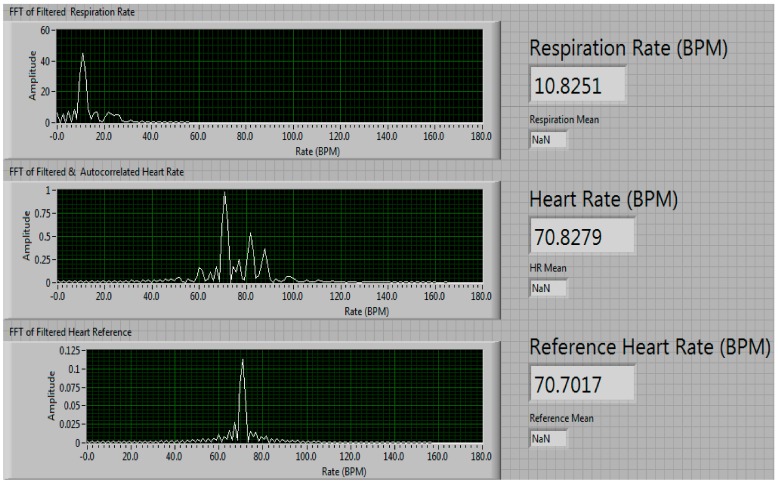
Frequency spectrum of vital signs signals after filtering and autocorrelation.

**Figure 7:**
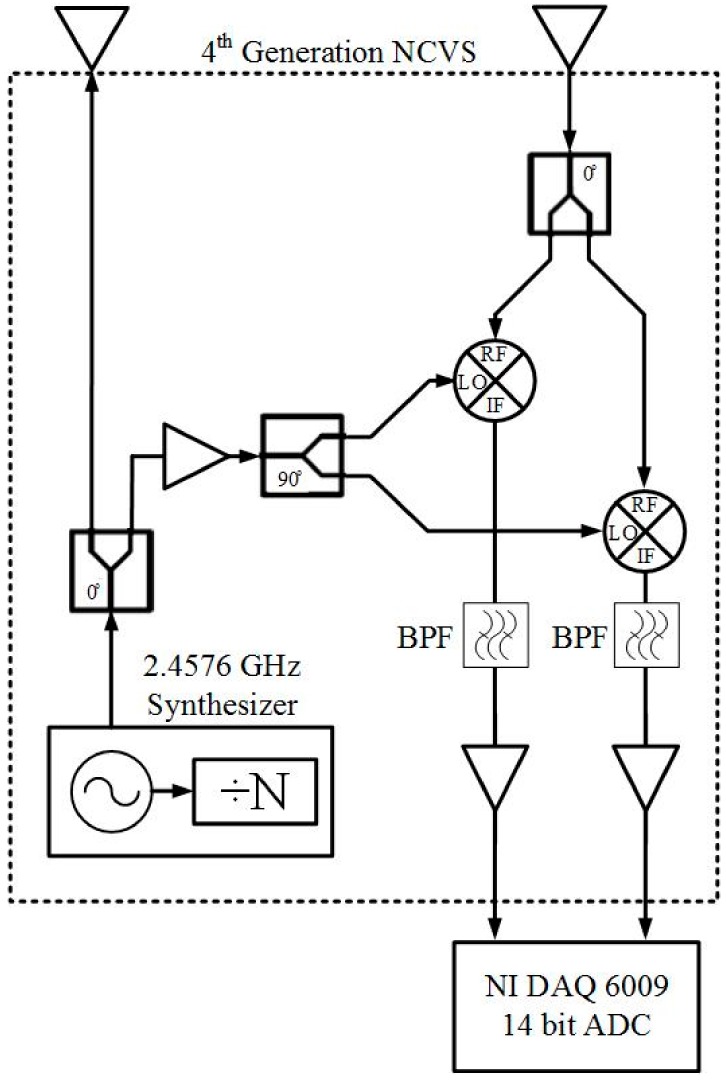
Block diagram of our 4th generation NCVS sensor.

Figure 3, Figure 4, Figure 5, Figure 6 and Figure 7 show the vital signs signals in the LabVIEW program. Figure 3 shows the in-phase and quadrature signals acquired by the ADC (blue), the piezoelectric reference sensor (red) and the arctangent demodulated signal (yellow). The reference signal is an external system that is not considered part of the NCVS sensor as it is only used to determine the heart rate accuracy of the NCVS as a reference.

Figure 4 shows the split arctangent signals after being filtered. The top graph shows the respiration rate signal after the high frequencies (i.e., the heart rate frequencies) have been filtered, the middle graph shows the heart rate signal after the lower frequencies (i.e., respiration frequencies) have been filtered and the bottom graph shows the reference sensor signal after using the same bandpass filter as the heart rate signal. Figure 5 shows all three signals after being run through an autocorrelation function. The autocorrelation of a signal shows how similar it is to itself at different times, which results in a measure of the signal’s periodicity. Through autocorrelation, the periodic nature of the signals is strengthened while the noise within the signal is reduced. Figure 6 shows all three signals after being transformed into the frequency domain using an FFT function. The numbers to the right of the graphs in Figure 6 shows the results of the peak detector function. In this instance, our NCVS shows the respiration rate measures 10.825 breaths-per-minute and the heart rate of 70.823 beats-per-minute (BPM). The piezoelectric reference sensor, which had the same signal processing performed as the NCVS signal, measured 70.702 BPM, just 0.1 BPM away from the heart rate our NCVS sensor measured.

### 2.2. A Brief Development History of Our Phased Array NCVS Sensor Systems

Over the past few years, there have been five generations of the NCVS sensors that we have developed in Professor. Donald Lie’s RF/Analog Soc Labs at Texas Tech. The first-generation system used Mini-Circuits RF modules and was designed as a proof-of-concept for vital signs extraction using a single-channel Doppler transceiver. This first-generation system used patch antennas for its transmitter and receiver antennas. The second generation of the NCVS sensor replaced the bulkier RF modules with surface mount components on an RF PCB and adopted an In-Phase and Quadrature (I/Q) Doppler transceiver design to reduce sensor position sensitivity by eliminate “null” points. The second-generation system replaced the patch antennas with Yagi-Uda PCB antennas for better directivity. The third-generation systems were a redesign of the second-generation system using updated components improved performance. However, the third generation NCVS sensor still did not include a phased-array beam-steering capability and had a shortcoming of temperature sensitivity on the frequency variation, so a fourth-generation board was designed.

**Figure 8:**
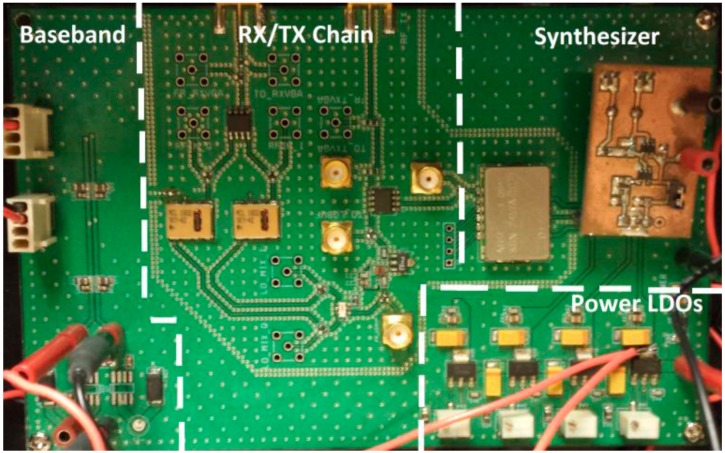
The PCB of our 4th Generation NCVS sensor.

The fourth generation NCVS sensor alleviated the issues of the third-generation system and replaced the VCO (voltage—controlled oscillator) with a frequency synthesizer to combat the frequency instability and high phase noise. This system was also tested with different antenna types, including patch, Yagi-Uda, log-periodic and custom made helical, to determine which antenna would work best for noncontact vital signs measurement. Figure 7 shows the functional block diagram of the fourth generation NCVS sensor and Figure 8 shows the populated PCB of the fourth-generation NCVS sensor.

**Figure 9:**
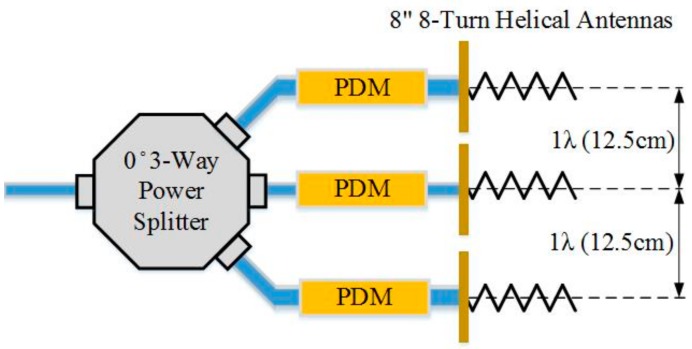
Our custom phased-array NCVS sensor system using 8” 8-turn helical antennas.

**Figure 10:**
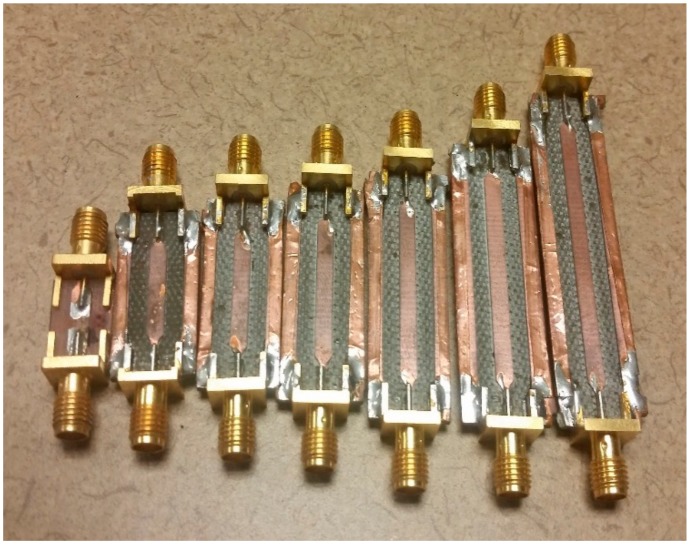
Example of a few phase delay modules (PDMs) used in our phased-array NCVS sensor.

After testing, it was determined that the custom made, 8”, 8-turn helical antennas performed the best for the accuracies of non-contact vital signs measurements due to its high directivity and also well-predicted behaviors of simulation vs. measured data for phased-array beam-steering [18]. Therefore, a phased array system using three custom-made helical antennas, shown in Figure 9, was implemented to render beam-forming for the 4th generation NCVS system and allow beam-steering without needed to physically move the antennas. The phased array system used a set of physical phase delay modules (PDM), pictured in Figure 10, to steer the beam as well as decrease the half-power beam-width (HPBW) of the system. Seven steering angle conditions, shown in Table 1 were chosen to the system to sweep across a nearly 30° range (14.1° left to 13.3° right).

**Figure 11:**
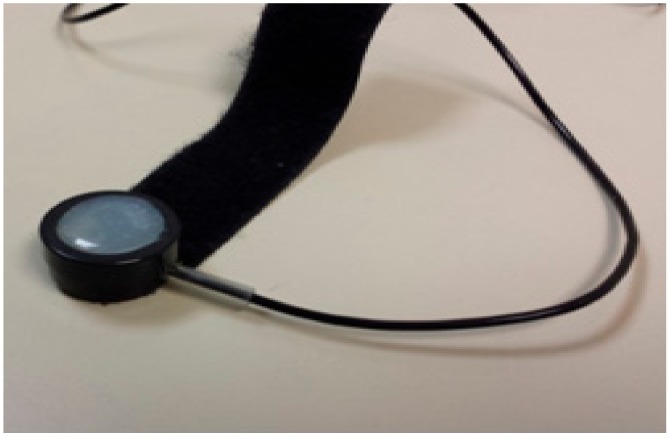
Piezoelectric finger sensor as a reference to determine NCVS sensor accuracy.

A piezoelectric finger sensor, shown in Figure 11, is used as a heart rate reference sensor to determine the accuracy performance of our NCVS sensor systems. The fourth generation phased-array system is the system used for the majority of the measurements showed in this paper, while we also made considerable updates and innovation to have created the 5th generation phased-array NCVS sensor system as well, with testing data shown in Section 3.3.

## 3. Testing of the Phased Array NCVS Monitoring System

A series of tests were performed using our fourth and fifth generation NCVS sensor systems within our labs. In the next sections, we will discuss our test setup and the results we gathered from these tests.

### 3.1. Robust Phased Array NCVS Monitoring in an Office Cubicle Setting

**Figure 12:**
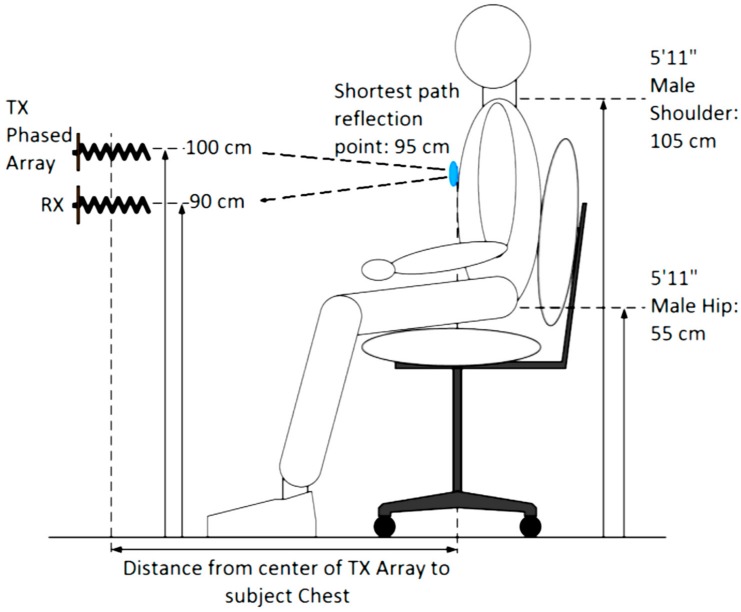
Diagram of our phased array NCVS sensor placement and the subject configuration for the evaluation of the horizontal phased array measurements.

**Figure 13:**
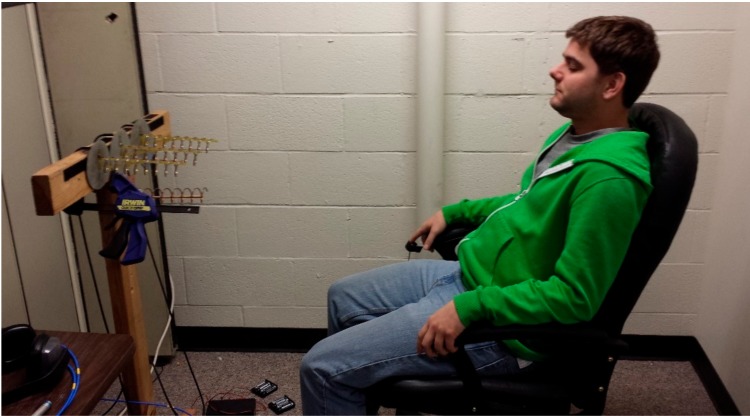
Subject (a student volunteer) monitored by our phased array NCVS sensor during a typical vital signs measurement setting.

**Figure 14:**
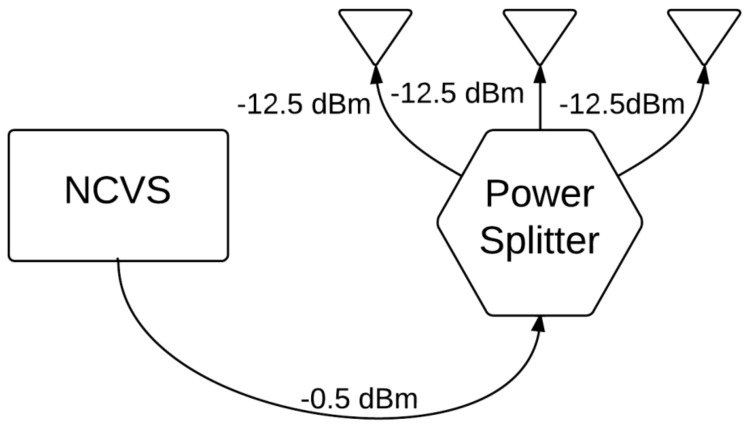
Phased array NCVS setup functional diagram of NCVS sensor with transmitting antenna port power of −12.5 dBm.

A key step taken to methodologically characterize our phased array NCVS sensor was to move the system out of the clutter-free anechoic chamber and into an office cubicle to determine the system’s effectiveness and robustness evaluated in a real-world office monitoring setting. These tests were all conducted within a Herman-Miller style cubicle using the fourth-generation NCVS sensor system and the piezoelectric reference finger sensor. Figure 12 and Figure 13 show the test setup used while testing the NCVS sensor inside the office cubicle setting, where Figure 1 plots the transmit (TX) antenna array (x3) is placed horizontally at a distance of 1 m away from the chest of the monitored subject. Only 1 receive (RX) antenna is used and it is placed right below the center of the TX array. Figure 14 shows the system diagram of the NCVS sensor during the first tests. For the first set of tests, the NCVS sensor was used with an output transmission power of −12.5 dBm going into each of the phased-array antenna port.

Upon evaluating the performance of the NCVS sensor, it appears that by increasing the transmission output power to +16.25 dBm the effective measurement distance can be extended to be around 200 cm. Table 2 shows a summary of all the distance measurements taken, where the IQR is the interquartile range, the difference between the upper and lower quartile.

### 3.2. Testing the Phase Delay Selection Board and Automatic Beam Steering Algorithm for Our 5th Generation Phased Array NCVS Sensor

When testing the phase delay selection board and the automatic beam steering algorithm, since one of the key application scenarios is intended for a long-term continuous NCVS monitoring during sleep, we need to change the orientation of the NCVS sensor and the subject’s position to test more data. The NCVS sensor’s orientation was rotated from a horizontal orientation to a vertical orientation. Subject’s positions were moved from a vertical orientation, sitting in a chair, to a horizontal orientation, lying on top of cushions on the ground. This orientation adjustment will enable better emulation of the kind of measurements that would be taken during overnight monitoring on a hospital bed or at home. As mentioned in the Introduction, we also made significant updates to enable the NCVS sensor system to perform automatic beam-steering with size reduction in creating the 5th generation phased-array NCVS sensor system for long-term NCVS monitoring [19]. The testing data of the 5th generation phased-array NCVS sensor with automatic beam-steering will be shown in Section 3.3.

**Figure 15:**
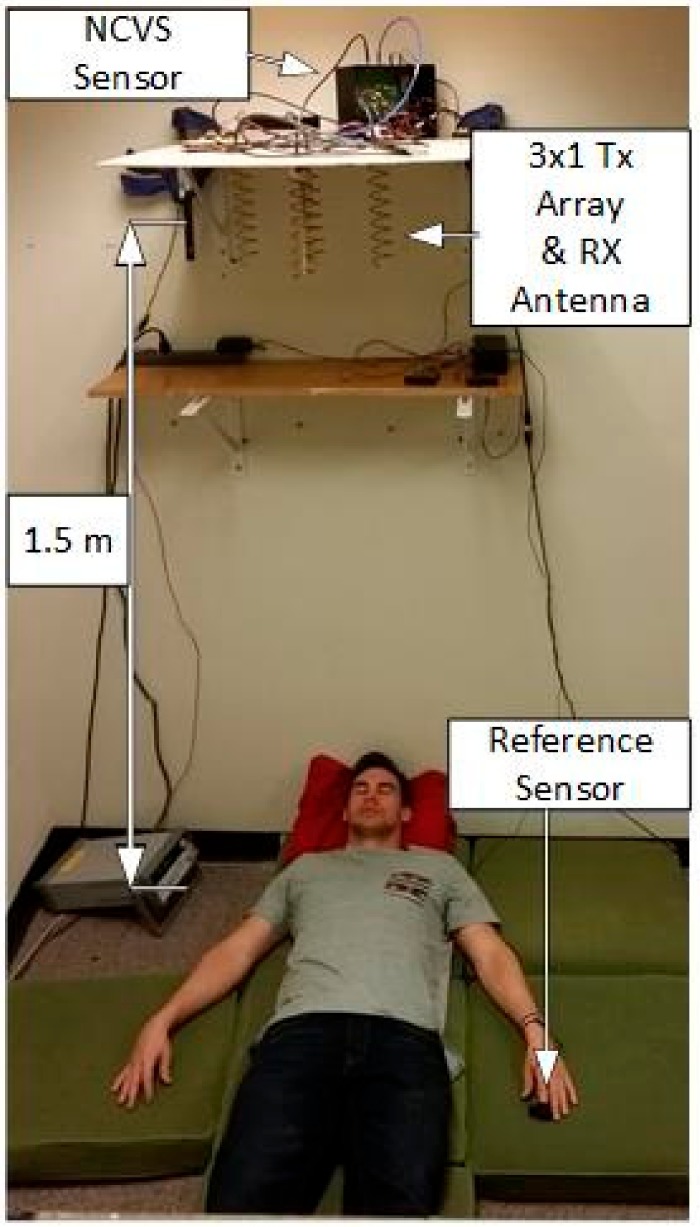
A test subject volunteer lying underneath our 5th Generation phased-array NCVS sensor inside a Herman-Miller cubicle using a vertical measurement orientation.

**Figure 16:**
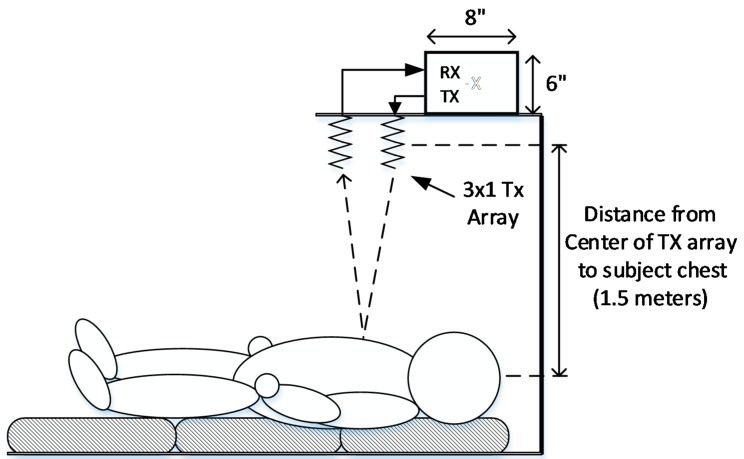
Vertical phased-array NCVS sensor test setup.

**Figure 17:**
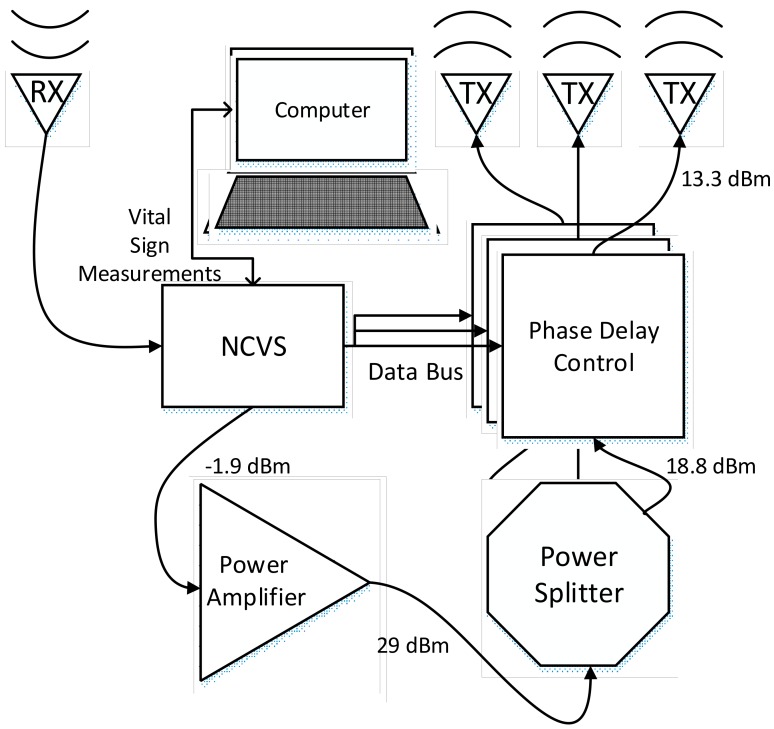
Our 5th-Generation phased array NCVS sensor on a single PCB: block diagram with the Phase Delay Control System for automatic beam-steering [19].

**Figure 18:**
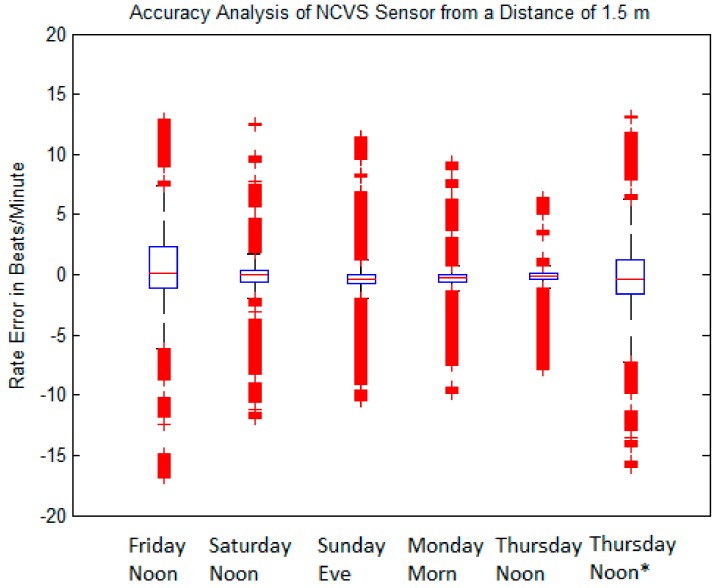
Boxplot of measurement errors for our phased-array NCVS system over various days of the week in a typical cubicle setting (subject lay down at 1.5 m away).

**Figure 19:**
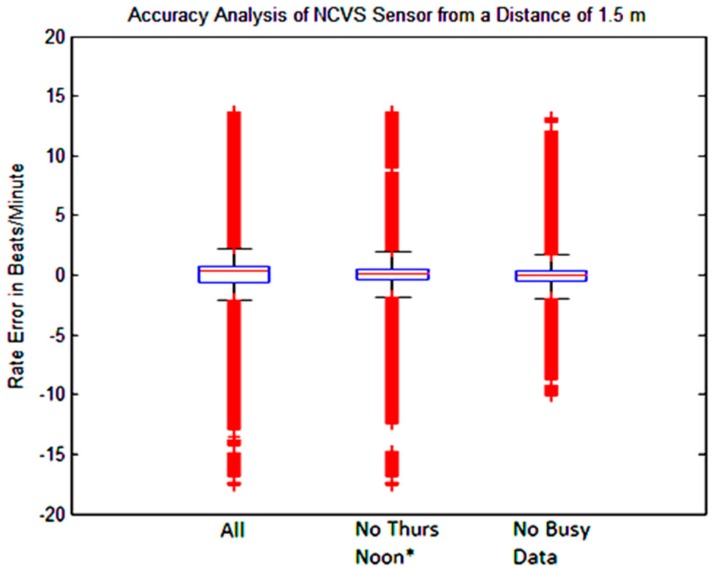
Boxplot of combined measurement error for our NCVS system over various days of the week in a typical cubicle setting (subject at 1.5 m away).

On our more integrated 5th generation NCVS system, we have replaced the physical phase delay modules (PDMs) and integrated them on the single PCB and developed an algorithm to control it, enabling a “smart” automatic vital sign measuring system that is capable of long-term non-contact vital signs monitoring. Figure 15 and Figure 16 show the new test setup used for evaluating the 5th generation phased-array NCVS sensor. Figure 17 shows the block diagram of the NCVS sensor measurement setup using the Phase Delay Control System for automatic beam-steering. The phase delay selection system and algorithm increases the accuracy of NCVS sensor by choosing the proper measurement angle to take vital signs readings at different locations as the subject moves. A quick measurement is taken initially at each possible angle and the angle with the highest (standard deviation)/(median ratio) is chosen for the data presented here. Once the received signal becomes too weak, the system will do an automatic re-sweep to track and find the best angle to resume monitoring. Note as the readers can see this very simple algorithm already achieved very good accuracy for our NCVS monitoring (e.g., see Figure 18 and Figure 19). We are still in the process of further improving this automatic beam-steering algorithm for long-term continuous NCVS monitoring with more testing subjects and will be publishing more sophisticated algorithms later. 

### 3.3. Long-Term Measurement Performance of Our Phased-Array Sensor

Now we are ready to perform more long-term NCVS monitoring. To determine the effectiveness of our NCVS system for long-term monitoring, we took a series of 20-min tests on different days of the week at various times. We have taken twenty-four 20-min tests (30,000 sample each; equivalent to a total of 8 h monitoring) for a total of 720,000 data points. Some tests were taken during active office hours; others were taken over the less active times. Figure 18 and Table 3 show the statistical analysis of the long-term NCVS testing data.

When observing the analyzed data across the various days of the week, it can be seen that the first dataset (“Friday Noon”) and the last dataset (“Thursday Noon *”) have lower accuracies than those of the other days of the week. This was because the first dataset (“Friday afternoon”) measurements were taken during a very busy time in the office. At that time, there was a meeting in a nearby cubicle. Consequently, because of the movement around the NCVS system and throughout the office, the accuracy was lower than average. In an attempt to understand this data better, we tried to recreate the background high noise level of that busy Friday noon time; therefore, we took an additional measurement the next Thursday afternoon as the last dataset (labeled “Thursday Noon *”), while deliberately walking around the NCVS system and the nearby cubicle areas in the similar manner as that “Friday Noon” measurement. Indeed, the “Thursday Noon *” results were rather close to the first dataset taken on the busy Friday noon time, leading us to believe that the current 5th Generation phased array NCVS sensor with the motion artifacts re-sweep algorithm is still not immune to the background movements in a busy office setting when heart rates are monitored at a distance of 1.5 m away. However, when our automatic phased-array NCVS sensor is used in a quieter environment (such as when the office is not so busy, or we suspect, in a private setting to monitor vital signs at home), the NCVS system can achieve about 94% accuracy of the monitored heart rate and the subjects would not need to remain stationary (see Table 4). Figure 18 and Table 4 show combined data of the measurements of all days, all data with the exception of the simulated noisy day and all data with the exception of the two noisy days.

Figure 19 shows the boxplot of the data in Table 4. It illustrates when heavy background movements are present, more data outliers appeared, probably due to larger background noise. It is important to note Table 4 indicates that even including the data taken during busy office hours, our automatic beam-steering 5th Generation phased-array NCVS system can still achieve over 90% monitoring accuracy on the heart rate signal (i.e., within ± 5 BPM), demonstrating the effectiveness of our phased array NCVS system for long-term continuous non-contact vital signs monitoring.

## 4. Conclusions

We have presented a mini-review on the design of non-contact sensors for long-term continuous vital signs monitoring, with the focus on our design prototypes of intelligent phased-array Doppler sensors that can perform automatic beam-steering to track the subject to enable long-term continuous heart and respiration rates monitoring. We have performed measurement data with statistics to show our Doppler NCVS sensor performed outside of the anechoic in a typical office cubicle setting and how its output power and background noise will affect the heart rate monitoring accuracy at different distances. It appeared that the NCVS sensor accuracy can be dependent on either the subject’s location to the beam angle, or even possibly depended on individual subjects themselves. We have, however, demonstrated the performance of our Doppler-based NCVS biosensor capable of taking accurate readings up to 2 m in an office cubicle setting with data from a handful of student volunteers. While our NCVS sensor is susceptible to background noise, the sensor is still capable of accurately measuring heart rate 90% of the time within 5 BPM of a reference sensor.

In order to move this NCVS system into clinical trials, we are working towards reducing the antenna size of our future generation NCVS system to be also considerably smaller and more portable. We are also taking more over-night monitoring data to check and improve on the NCVS system’s automatic steering and tracking algorithms. We believe our NCVS sensor system can be a robust, automatic, intelligent long-term vital-signs monitoring tool that has clinical advantages and applications. More long-term testing data is needed to quantitatively analyze the effects of the background noise and the subject’s natural movement during sleep on the accuracy and reliability of overnight vital signs monitoring. We will report our findings on these important topics with statistical data in the near future. Subsequently, we have planned to seek TTUHSC IRB ((Internal Review Board) approval to perform clinic trials of long-term NCVS monitoring on patients during their sleep for detecting/preventing conditions such as sleep apnea, sudden infant death (SID), sudden cardiac arrest (SCA) and other applications.

## Figures and Tables

**Table 1:** Phased-array beam steering conditions used in our NCVS sensor.

Condition	Delay 1	Delay 2	Delay 3	Measured Steering Angle	Direction
**1**	0°	90°	180°	13.3°	Right
**2**	0°	60°	120°	9.9°	Right
**3**	0°	20°	40°	3.9°	Right
**4**	0°	0°	0°	0°	Straight
**5**	40°	20°	0°	−4.6°	Left
**6**	120°	60°	0°	−10.1°	Left
**7**	180°	90°	0°	−14.1°	Left

**Table 2:** NCVS sensor performance at various ranges and output powers (at 0° steering angle).

P_OUT_ (at Each TX Antenna Port)	Distance from Subject	50 cm	75 cm	100 cm	150 cm	200 cm
−12.5 dBm	Within 1 bpm	83.7%	78.0%	40.2%		
	Within 2 bpm	88.8%	83.3%	42.9%		
	Within 5 bpm	93.0%	91.4%	51.80%		
	IQR	0.45	0.62	19.8		
	STDEV	4.81	3.91	13.25		
9.7 dBm	Within 1 bpm		92.7%	86.2%	80.3%	30.8%
	Within 2 bpm		94.4%	89.4%	85.3%	37.4%
	Within 5 bpm		95.9%	93.2%	89.9%	48.1%
	IQR		0.33	0.44	0.66	12.9
	STDEV		2.06	3.56	4.05	12.2
16.25 dBm	Within 1 bpm		84.9%	84.8%	85.5%	81.8%
	Within 2 bpm		89.8%	88.0%	88.8%	86.60
	Within 5 bpm		93.3%	92.3%	93.6%	91.8%
	IQR		0.5	0.47	0.49	0.64
	STDEV		2.86	3.52	2.74	4.96

**Table 3:** Measured vital signs statistics using our phased-array NCVS sensor over various days of the week.

	Friday Noon	Saturday Noon	Sunday Eve	Monday Morn	Thursday Noon	Thursday Noon *
**1 BPM**	69.89	89.16	89.03	88.86	87.22	*75.42*
**2 BPM**	76.60	92.19	92.66	91.99	89.22	*79.40*
**5 BPM**	83.46	93.69	95.23	93.82	92.86	*82.34*
**IQR**	1.433	0.647	0.602	0.505	0.610	*1.448*
**STDEV**	4.306	2.419	2.134	1.941	1.821	*4.288*

**Table 4:** Measured vital sign statistics using our phased array NCVS sensor system over various days. The “Busy Data” refers to the data taken during the two days when lots of background movement noises were present.

	All Data	No Thurs. Noon *	No “*Busy Data*”
**1 BPM**	83.26	84.83	88.57
**2 BPM**	87.01	88.53	91.51
**5 BPM**	90.23	91.81	93.90
**IQR**	0.87	0.759	0.591
**STDEV**	2.82	2.524	2.079
